# On the Antiquity of Cancer: Evidence for Metastatic Carcinoma in a Young Man from Ancient Nubia (*c.* 1200BC)

**DOI:** 10.1371/journal.pone.0090924

**Published:** 2014-03-17

**Authors:** Michaela Binder, Charlotte Roberts, Neal Spencer, Daniel Antoine, Caroline Cartwright

**Affiliations:** 1 Department of Archaeology, Durham University, Durham, United Kingdom; 2 Department of Ancient Egypt and Sudan, British Museum, London, United Kingdom; 3 Department of Conservation and Scientific Research, British Museum, London, United Kingdom; University of Oxford, United Kingdom

## Abstract

Cancer, one of the world’s leading causes of death today, remains almost absent relative to other pathological conditions, in the archaeological record, giving rise to the conclusion that the disease is mainly a product of modern living and increased longevity. This paper presents a male, young-adult individual from the archaeological site of Amara West in northern Sudan (*c*. 1200BC) displaying multiple, mainly osteolytic, lesions on the vertebrae, ribs, sternum, clavicles, scapulae, pelvis, and humeral and femoral heads. Following radiographic, microscopic and scanning electron microscopic (SEM) imaging of the lesions, and a consideration of differential diagnoses, a diagnosis of metastatic carcinoma secondary to an unknown soft tissue cancer is suggested. This represents the earliest complete example in the world of a human who suffered metastatic cancer to date. The study further draws its strength from modern analytical techniques applied to differential diagnoses and the fact that it is firmly rooted within a well-documented archaeological and historical context, thus providing new insights into the history and antiquity of the disease as well as its underlying causes and progression.

## Introduction

Today, cancer represents one of the leading causes of death worldwide [1], with numbers more than doubling over the past thirty years. It is a particular feature of the 2^nd^ epidemiological transition where industrialisation developed, living conditions improved, there was a decline in mortality from infectious diseases, and a corresponding rise in chronic non-infectious diseases such as cancer. Cancer’s global increase has largely been blamed on environmental and lifestyle related factors such as smoking, dietary constituents and pollution, as well as a longer life expectancy [1], [2], [3]. While the world now finds itself in the 3^rd^ epidemiological transition (the re-emergence of infectious diseases and new infections along with resistance to antibiotics), our human population has also experienced a transition to agriculturally based societies from hunting and gathering (1^st^ transition). At each transition it is well known that socioeconomic, political, and environmental factors all contribute to health and well-being, but also ill health [4].

However, very little is known about the antiquity, epidemiology and evolution of cancer in past human populations. Nevertheless, ancient medical documents indicate pathological conditions, tentatively identified as cancer, were known both to the Ancient Egyptians and Greeks [5], [6]. The Edwin Smith Papyrus (*c*. 1600BC but assumed to be a copy of a document dating to *c.* 3000BC) provides the earliest known reference to a tumor-like swelling of the breast [7], [8] and is generally believed to be also the earliest known description of cancer *per se* [9].

Increasingly, evolutionary approaches are being taken to the understanding many health problems today, cancer being one [10], [11]. It has also been highlighted that of all other species, humans are more likely to contract cancer because we live a lot longer, especially now, and humans are challenged with biologically adapting to rapidly changing factors that were introduced in the 1^st^ and 2^nd^ epidemiological transitions. These include a changing diet with high sugar and fat content, increased alcohol and tobacco consumption, and environmental pollutants. Furthermore, it has been noted that a genetic predisposition may increase a person’s risk of cancer [12] and that pathogens can be important in the development of cancer. This has implications for the many co-morbidities seen today, but one of the key features of the 3^rd^ epidemiological transition, an increase in infectious disease due to newly emerging and re-emerging infectious disease in combination with a resistance to antibiotic therapy (and cancer therapies), is particularly relevant for this paper [13]. Whilst understanding this evolutionary framework for cancer, and in spite of a long history of palaeopathological study of human remains globally [14], the direct evidence of cancer from ancient human remains is still very rare. This remains the case despite the constantly growing number of remains available for study, and an increase in numbers of bioarchaeologists. This makes the rigorous study of early evidence for cancer particularly important if palaeopathology is to contribute to a better understanding of its evolution and increasing presence today.

While primary bone cancer is very rare even in modern populations, secondary skeletal involvement due to metastatic spread of a soft tissue cancer is very common [15]. Consequently, the dearth of evidence from ancient skeletons has led to the common conception that cancer was very rare in antiquity [3]. This is usually explained by two main factors: shorter life spans and a healthier living environment. Even though the underlying pathological processes of cancer are still far from being fully understood, it is clear that mutations during growth and division of cells represent the initial step in cancer genesis [16]. With increasing age, the risk of mutations naturally increases as the reproductive cycle of cells becomes more prone to error. Therefore, the more common types of malignant cancer, particularly those causing secondary bone involvement, show a predilection for older age ranges [17]. Prior to the onset of modern medical care and improved sanitary conditions, average life expectancies are assumed not to have exceeded 30-50 years due to infectious diseases [18]. People in the past simply did not, in general live long enough to develop cancer [19]. The rise in cancer prevalence today therefore is seen as a consequence the 2^nd^ epidemiological transition when significantly higher life expectancies were experienced [20]. However, it has been recognised that this absence of old individuals in past populations may in fact be a misconception created by inadequate methods for accurately estimating adult age-at-death in human remains, particularly with regard to older age groups [21], [22]. Textual evidence from the Egyptian New Kingdom (*c*. 1500-1070BC) [23] or Roman period [24] does, however, provide ample evidence that some individuals did indeed live into their 60s and 70s. Other types of cancer, including most primary bone cancers, predilect for younger ages [17], but these are almost completely absent from the palaeopathological literature [25].

The second explanation for the apparent absence of cancer in antiquity is related to the fact that the main causes for cancer, estimated to account for up to 80% of cancer-related deaths today, are associated with a modern life style such as smoking, dietary habits, and a lack of physical activity [26]. The sharp increase of palaeopathological cancer evidence over the past centuries in the wake of increasingly modern living conditions provides ample support for this claim.

### Palaeopathological evidence of malignancies

To date, only around 200 skeletons and mummified individuals from around the world have been reported with different primary and secondary malignancies [3], [25]. However, diagnosis of cancer in human remains is not straightforward [27]. Particularly if comprising lytic lesions, differentiation from other pathologies or post-depositional damage is not always possible upon macroscopic examination alone, and often requires additional analytical techniques such as radiography or SEM [27], [28]. Moreover, many palaeopathological reports of cancer derive from the early days of bioarchaeological research and diagnosis is often solely based on morphological appearance. Due to the often inadequate descriptions in publications, precluding a reliable re-evaluation of previous analyses, combined with the fact that many of these skeletons and mummies are not available for examination any more, the majority of these reports should be considered tentative at best [29]. Equally problematic is the fact that due to research and excavation strategies skeletal collections are often confined to skulls or selected pathological bones. Thus, in most early reports diagnosis is based on isolated skeletal elements, preventing an examination of the full range of pathological changes.

The earliest generally accepted example of a malignant neoplasm was reported in a Neolithic skeleton (*c.* 4000BC) from Austria displaying signs of multiple myeloma [30]. Further often cited early examples of malignant neoplasm are reported from the Czech Republic [31] and Russia [32] (Table 1); again, the lack of adequate publication leaves doubts as to their diagnosis. The vast majority of palaeopathological evidence only dates to the past 500 years of human history, whereas evidence for cancer before modern era remains very sporadic [25], [33], [34]. With regard to geographic distribution, the majority of evidence is from Europe and Egypt, undoubtedly biased by the large number of skeletal assemblages recovered from these areas. Nonetheless, examples are also known from Australia, North and South America [25].

**Table 1:** 

Site	Country	Dating	Diagnosis	Preservation	Site of involvement	Reference
*Primary bone tumors*						
Bassa Padana	Italy	Neolithic	osteosarcoma	skeletal	ulna	[93]
?	Egypt	1500-1070BC	osteosarcoma	skeletal	humerus**	[93]
Münsingen	Switzerland	800-600BC	osteosarcoma	skeletal	humerus	[19]
*Secondary bone tumors*						
Mauer	Austria	*c.* 4000BC	multiple myeloma	skeletal	skull	[94]
Giza	Egypt	3000BC	metastatic carcinoma – nasopharyngeal*	skeletal	skull**	[41]
Naga-ed-Deir	Egypt	2300-1800BC	metastatic carcinoma – nasopharyngeal	skeletal	skull**	[34], [95]
?	Czech Republic	2200-800BC	metastatic carcinoma*	skeletal	skull	[31]
?	Russia	1500BC	metastatic carcinoma*	skeletal	skull	[32]
Thebes West	Egypt	1500-500BC	3 individuals with of metastatic carcinoma, 2 with multiple myeloma	skeletal	various sites	[29]
Arzhan	Russia	700BC	metastatic carcinoma – prostate	skeletal	skull, axial skeleton, humerus, femur	[96]
?	Egypt	285–230BC	metastatic carcinoma – prostate	mummified	spine, sacrum	[42]
Abusir/Saqqara	Egypt	664-332BC	4 individuals with of metastatic carcinoma or multiple myeloma	skeletal	various sites	[94]

Early evidence of cancer (* indicates commonly cited but a dubious diagnosis or criticised in later publications, ** indicates isolated skeletal element, ? site unknown).

The relatively high number of reports of cancer in human remains from ancient Egypt when compared to the rest of the world cannot only be ascribed to the wealth of excellently preserved mummified and skeletal human remains but also its longstanding history of palaeopathological research [35]. To date, around 50 individuals with primary and secondary malignancies have been described in the literature [29], [34], [36], [37], [38], [39], [40], [41], [42] even though, again, reports are often unconvincing and inadequate publication makes their reassessment impossible [43].

The earliest Egyptian example is found on an Old Kingdom skull (*c*. 3000 BC) from Giza in Egypt [41]. Lytic lesions were identified as metastatic carcinoma, thought to originate from a nasopharyngeal tumor, though this diagnosis is disputed [44]. With four more tentative examples ranging in date between 2300BC and 300AD [34], [41], nasopharyngeal carcinoma is the most commonly reported type of cancer in Ancient Egyptian collections. A further 14 individuals with malignant primary and secondary neoplasm dated from the Archaic to New Kingdom periods (*c.* 3000-1000BC), are cited in the early palaeopathological literature [36], but again doubts remain as to their accurate diagnosis. More convincing, recently published early examples were found in tombs at Thebes (modern Luxor) dating to between 1500 and 500BC [29]. The vast majority however, again date from the 1^st^ millennium BC onwards. While most of the evidence so far comes from skeletal remains, evidence of soft tissue tumors from mummified remains, to date, very rare, to date [45]. Only recently, the first convincing evidence of prostate cancer was detected through computerised tomography of a Ptolemaic (285–230BC) mummy [42].

Despite the large number of skeletal human remains available, reports of malignancies from Nubia are rare up until now. One case of metastatic carcinoma was described in a Meroitic period male (350BC-350AD) from Wadi Halfa [46] and a second Meroitic individual has recently been identified at Sai Island [47].

### Amara West

**Figure 1:**
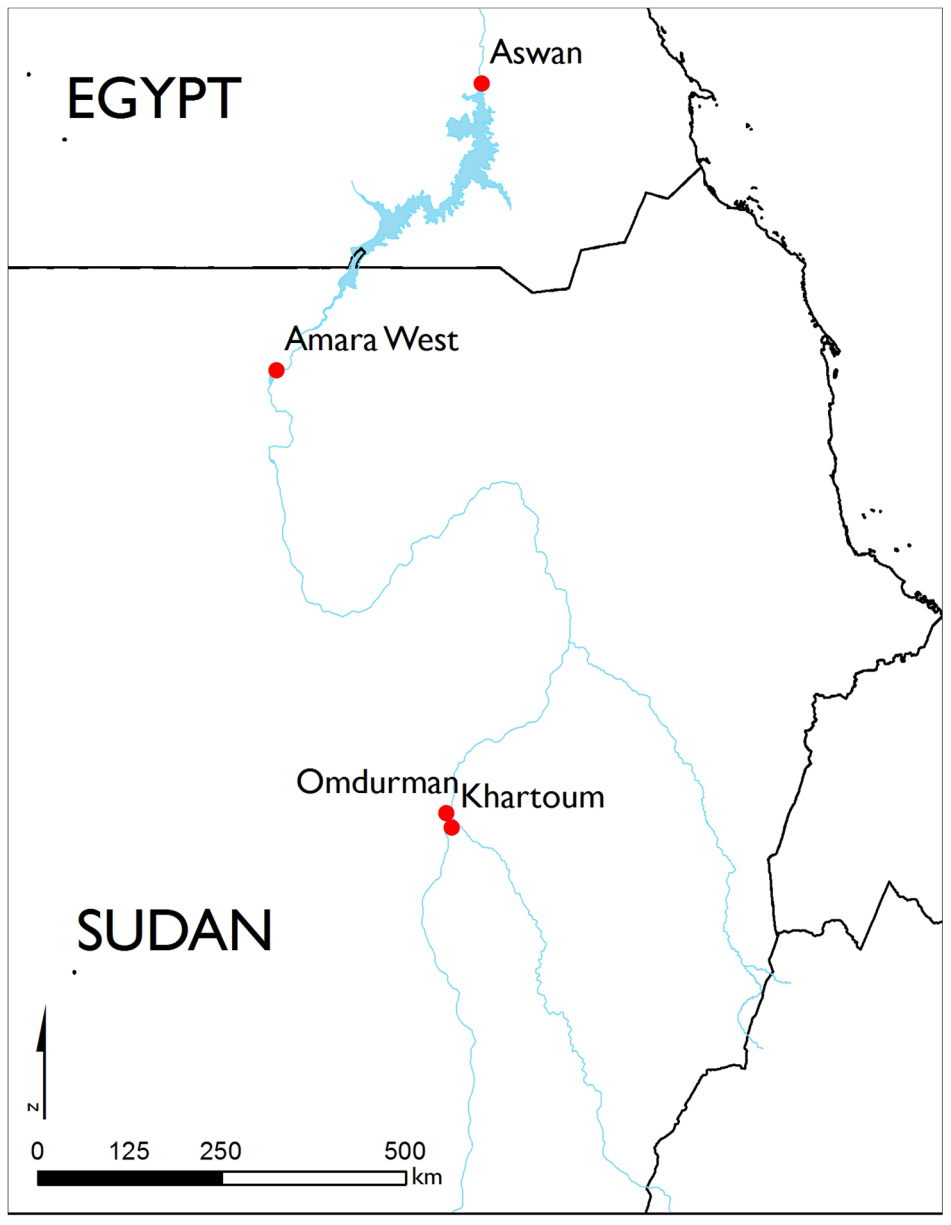
Location of Amara West. Map of modern Sudan showing the location of the archaeological site (Map drawn by M. Binder, source: ESRI).

This paper presents the human skeletal remains of an individual from the archaeological site of Amara West in modern Sudan, situated on the left bank of the Nile, 750km downstream of the country’s modern capital Khartoum (Figure 1). The settlement is understood to have been founded around 1300 BC as a new administrative capital for the region of Kush (Upper Nubia), on the basis of administrative titles inscribed within a formal building in the town [48]. The region had been controlled by the pharaonic state from around 1500 BC, through the construction of planned settlements. A British Museum research project, directed by Neal Spencer ( Department of Ancient Egypt and Sudan), is investigating the lived experience of the people buried within the ancient town [49], [50], through renewed excavations in both the town and its cemeteries [51], [52], complemented by a range of bioarchaeological and environmental analyses. Archaeological fieldwork has been undertaken by the British Museum since 2008, focussing on the walled town [49] and two associated cemeteries [51], [52]. The town’s occupation, which continues for several centuries after the pharaonic state lost control of Upper Nubia, coincided with a time of drastic environmental deterioration affecting the entire Nile valley region [53].

Individuals were being interred until around 800BC in the two cemeteries, on the basis of 14C dates (human bone and linen associated with the burials) and the ceramic assemblages. Archaeological and botanical evidence indicates a largely agricultural community with a subsistence based on grain cultivation and livestock [54], [55], although integrated into the trading framework of pharaonic Egypt, which includes, for example, the import of luxury Mycenaean pottery. Epigraphic evidence from the site [48] indicates the presence of a number of administrative officials and priests present in the town.

**Figure 2:**
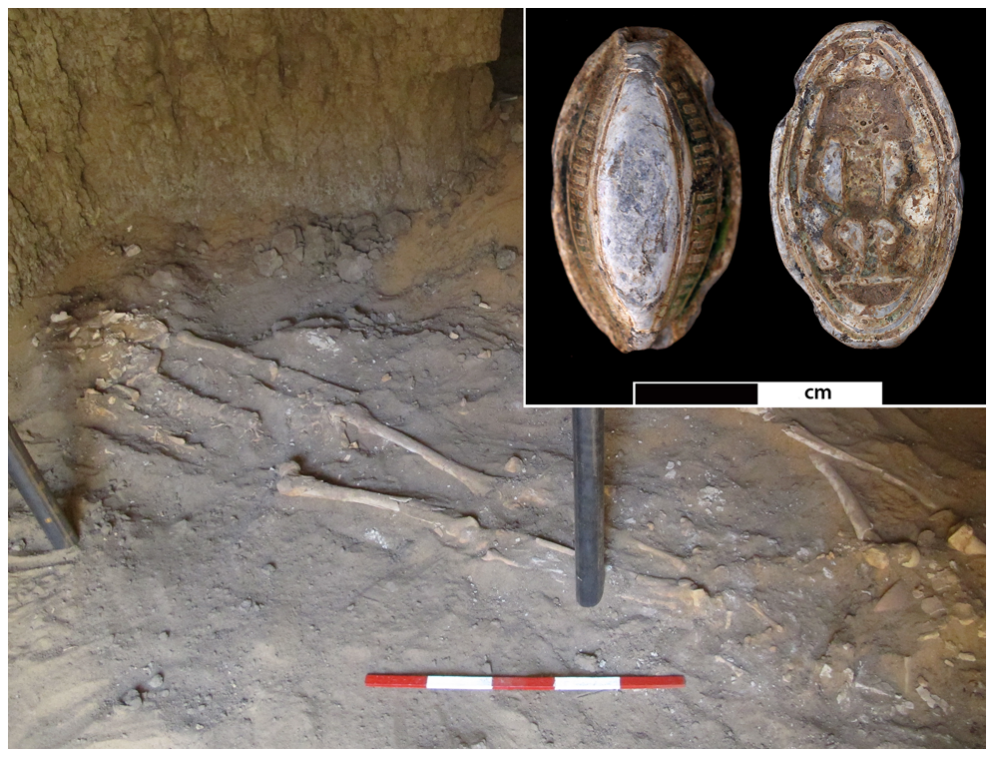
The skeleton Sk244-8. Skeleton Sk244-8 in its original burial position in the western chamber of G244. The insert shows faience amulet F9273 found associated with the individual from both sides. The Egyptian god Bes (right side) is depicted on the reverse side.

The individual (skeleton 244-8), discussed here was recovered in 2013 from tomb G244, located in the north-eastern cemetery (C) of Amara West. Based on tomb architecture and aspects of funerary ritual, this burial ground appears to have been used for the sub-elite population of the town [51]. In contrast, elite funerary monuments with pyramid superstructures are found in a second, contemporary, cemetery (D) on a desert escarpment to the north-west of the town [52]. Tomb G244 is marked by a substantial burial mound (tumulus), representing a distinctive hallmark feature of indigenous Nubian funerary customs. In contrast, its internal layout, comprising of five large underground burial chambers used for multiple burials, is entirely Egyptian in nature and consistent with contemporary examples in Egypt proper. As such, the tomb’s architecture attests to a unique, hybrid culture which developed in Amara West and similar settlements over the course of several episodes of Pharaonic domination over Nubia [56], [57]. Similar to the eight other burials in the first western chamber, individual 244-8 was buried extended, within a badly deteriorated painted wooden coffin, and provided with a faience scaraboid (Figure 2). The position of the skeletal elements suggests tight wrapping of the burial. Although there is no evidence to suggest the elite status of the individual, funerary architecture and the grave good assemblage do indicate a certain degree of wealth of the individuals buried in this tomb. The well preserved ceramic assemblage recovered from the tomb provides a date within the 20^th^ Dynasty (1187-1064BC).

## Methods

Skeleton 244-8 has been accessioned into the collection of the Department of Ancient Egypt and Sudan of the British Museum, London (EA 83132), through the generosity of the National Corporation for Antiquities & Museums (Sudan). All necessary permits were obtained for the described study, which complied with all relevant regulations. The skeletal remains were analysed applying standard anthropological and bioarchaeological methods [58], [59]. Estimation of sex was carried out based on morphological markers on the pelvis and skull [58], [60]. Age-at-death was estimated based on age-related changes in the pubic symphysis [61] and markers of skeletal maturation [62]. Examination of pathological changes was first carried out macroscopically and with the use of a hand lens whereby all detected abnormal lesions were described and mapped according to their anatomical region. All elements of the skeleton were investigated radiographically (Seifert Isovolt DS1 X-ray tube). Selected lesions were further examined using an digital microscope (DinoLite AM7013MT Premier) and through SEM (Hitachi S-3700N variable pressure scanning electron microscope). Radiography and SEM were both carried out within the facilities of the Department of Conservation & Scientific Research at the British Museum.

## Results

Sexual dimorphic features in the skull, mandible and pelvis suggest the individual is male. Age estimation, based on the pubic symphysis of the pelvis [61] as well as remnants of epiphyseal union in the distal tibiae visible upon radiographic examination, indicates an age at death between 25 and 35 years [62].

**Figure 3:**
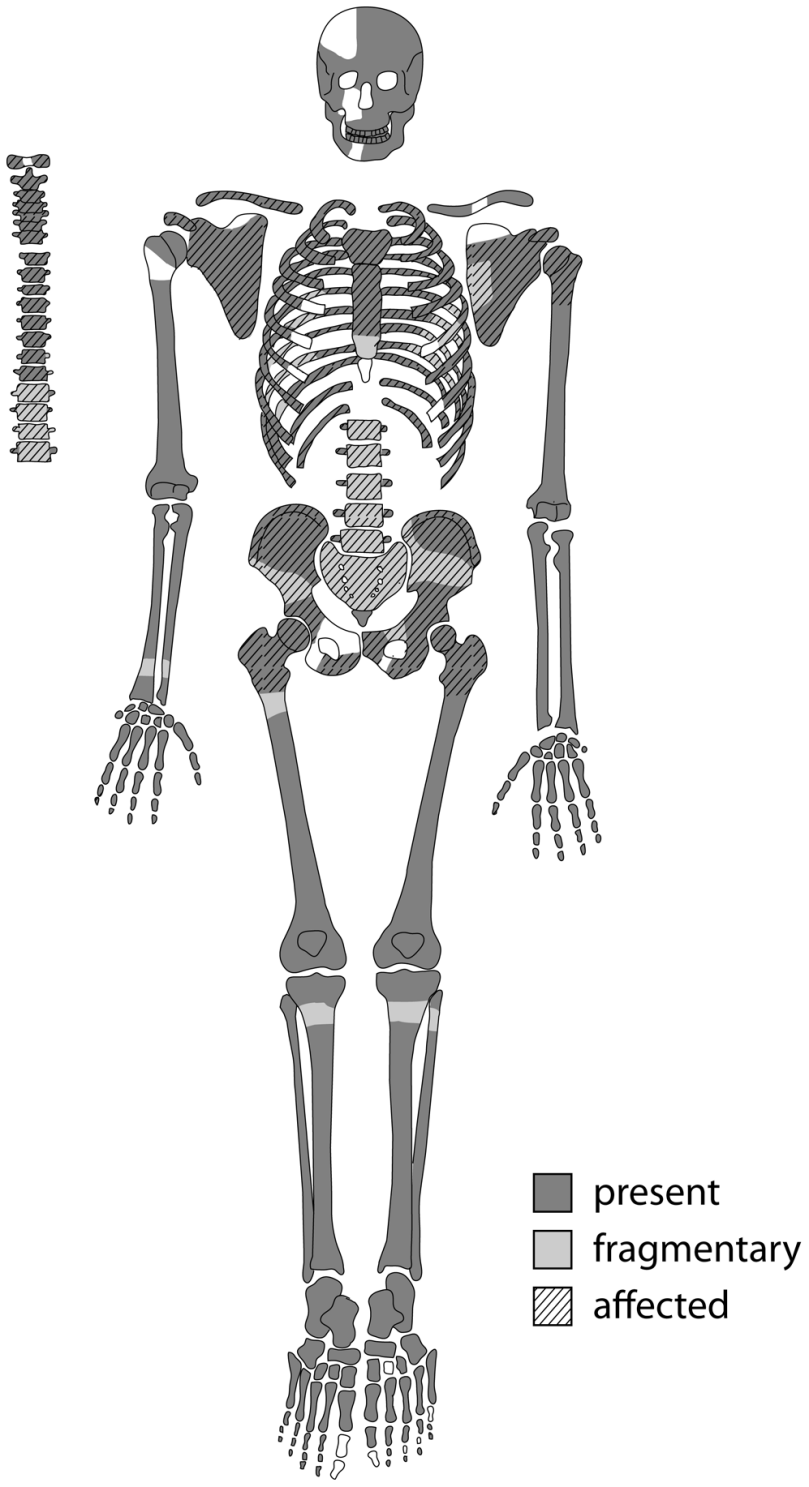
Preservation of Sk244-8. Preserved elements of the skeleton and elements affected by pathological changes. Dark areas indicate full preservation, light areas indicate fragmented areas. Hatched areas are the bones affected by lytic lesions.

The skeleton is almost complete with largely intact bone surfaces (Figure 3). The long bones and skull of the skeleton suffered little to moderate post-mortem breakage. In contrast, the elements of the axial skeleton are very friable and fragmentary due to the pathological conditions present, and are described below. Taphonomic damage, mainly due to salt precipitation from the surrounding soil, led to some erosion on the skull vault. A multitude of small round to oval-shaped osteolytic lesions ranging in size from between 3 and 30 mm in size are observable in the scapulae, clavicles, sternum, vertebrae and pelvis. In the skeletal elements with large amounts of cancellous bone, in particular the bodies of thoracic and lumbar vertebrae as well as the sacrum and pelvis, a high degree of fragmentation made detection and description of individual lesions difficult.

**Figure 4:**
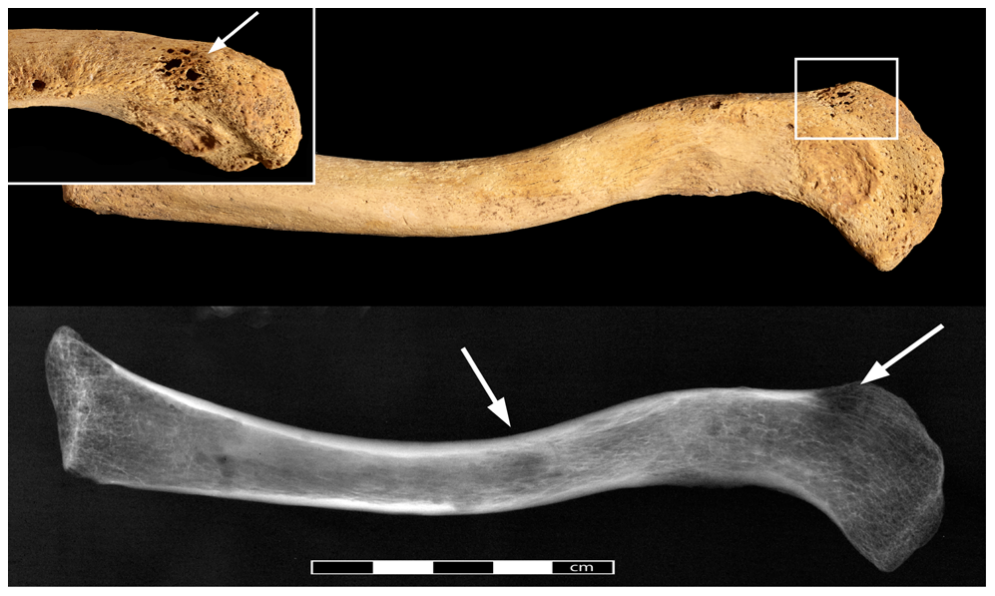
The left clavicle of Sk244-8. General view of the left clavicle with the pathological lesions indicated by arrows. Radiograph of the same bone on the bottom. Insert shows close-up of the lesion on the superior margin indicated by rectangle.

**Figure 5:**
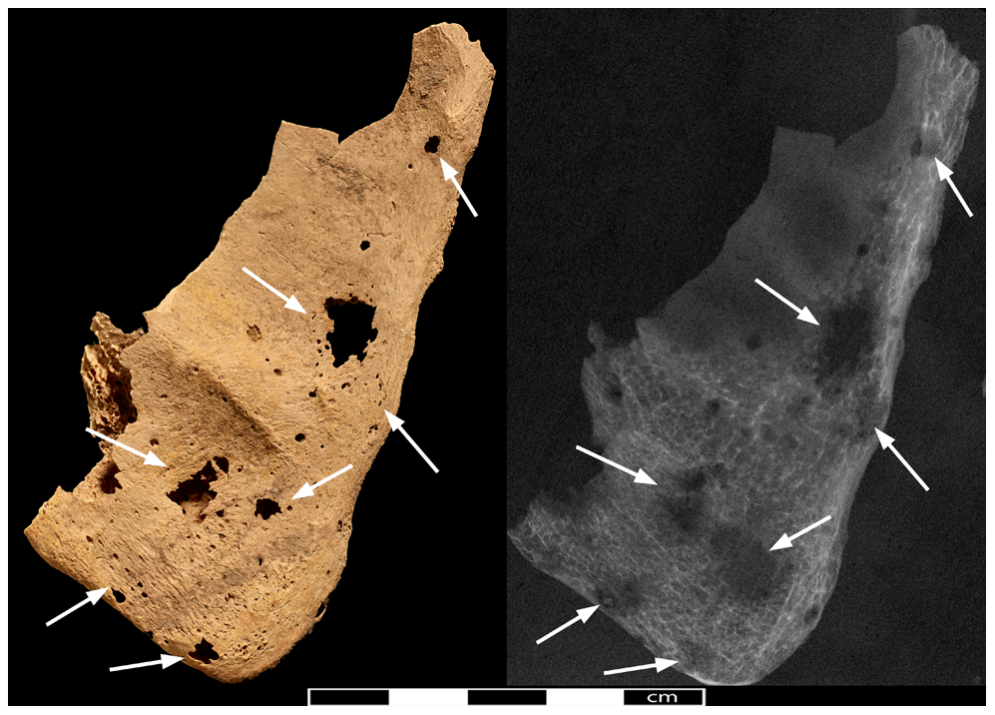
Pathological lesions in the right scapula. Photo- and radiograph of the right scapular blade (view from anterior, arrows indicate location of lytic lesions).

**Figure 6:**
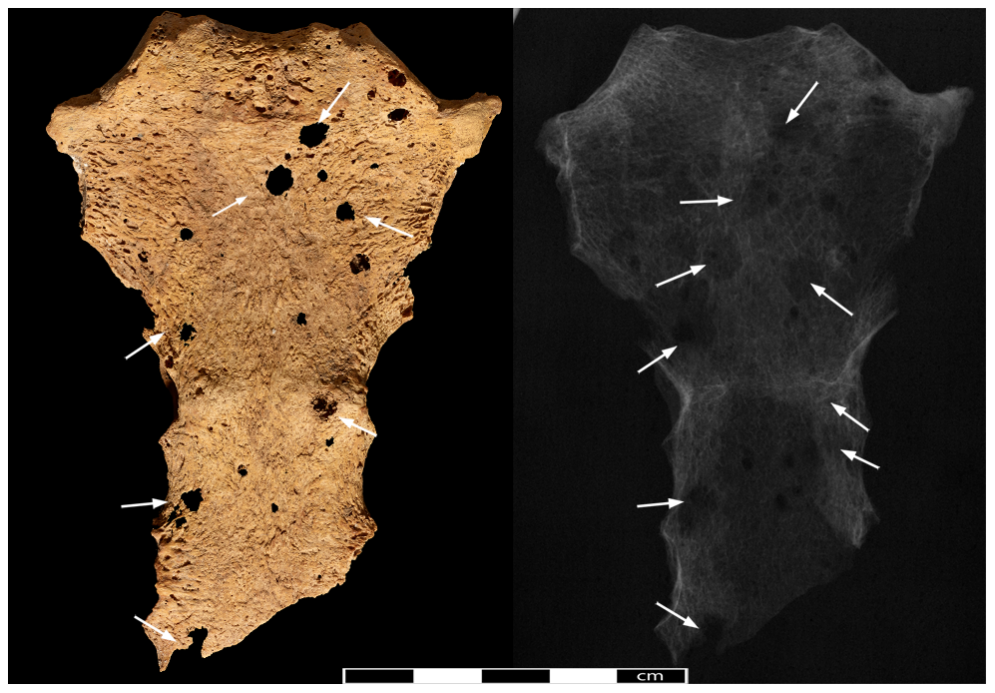
The sternum of Sk244-8. Anterior aspect of the sternum. Arrows indicate location of lytic lesions.

In the clavicles, pinpoint-sized perforations are only visible on the superior margin of the lateral end of the right clavicle (Figure 4). Upon radiographic examination, these lesions present themselves as a considerably larger sub-circular cavitation (9 mm). In addition, the radiograph shows a second clear circular lesion in the mid-shaft. Both scapulae display a multitude of round to irregular lesion. In the right scapula (Figure 5) three irregular, ovoid (10×4 mm, 12×7 mm, 10×7 mm) lesions affected the inferior half of the scapular blade, accompanied by smaller, round ones (1–3 mm) along the medial and lateral edges. The left side also displayed a clear round lesion (Ø 8 mm) on the coracoid process, two more (Ø 6 mm) extra-articular lesions on the inferior side of the glenoid cavity and one large irregularly shaped lesion on the medial edge of the inferior angle (4×5 mm).The sternum was affected by some post-depositional damage, particularly on the visceral side, making differentiation of pathological lesions difficult. Upon radiographic examination at least seven sub-circular lesions (7–28 mm) were observed within the cancellous bone (Figure 6). The left humeral head is intact, and several small post-depositional defects were present on the anterior side. The radiographic analysis of the left humeral head also revealed two distinctive radiotranslucent foci approximately 8 mm in diameter below the Glenoid joint surface and in the lesser trochanter.

**Figure 7:**
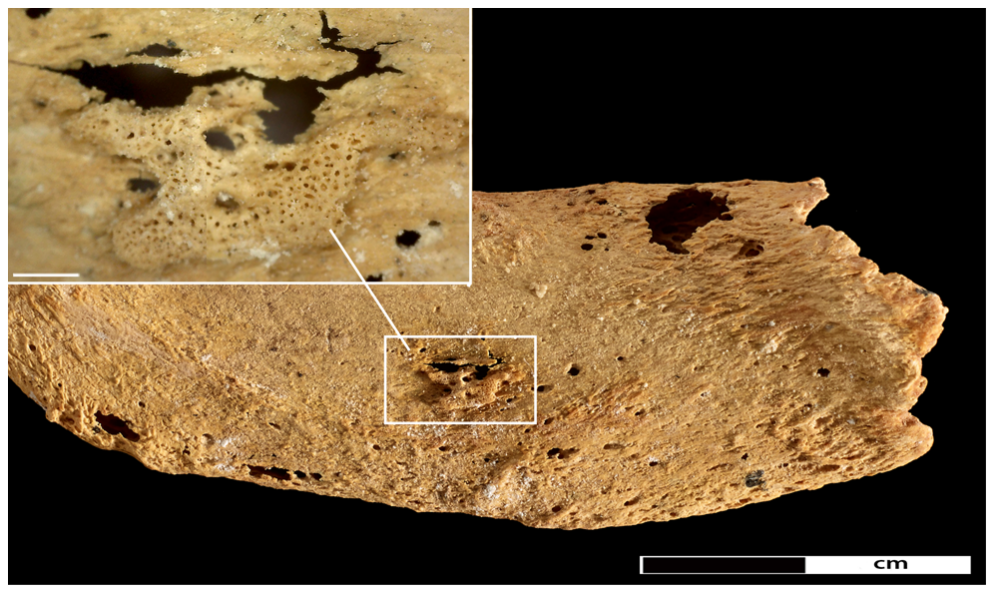
Pathological changes in the first rib. New bone formation on the inferior aspect of the left first rib. Insert shows close-up of area of new bone formation (45× magnification).

**Figure 8:**
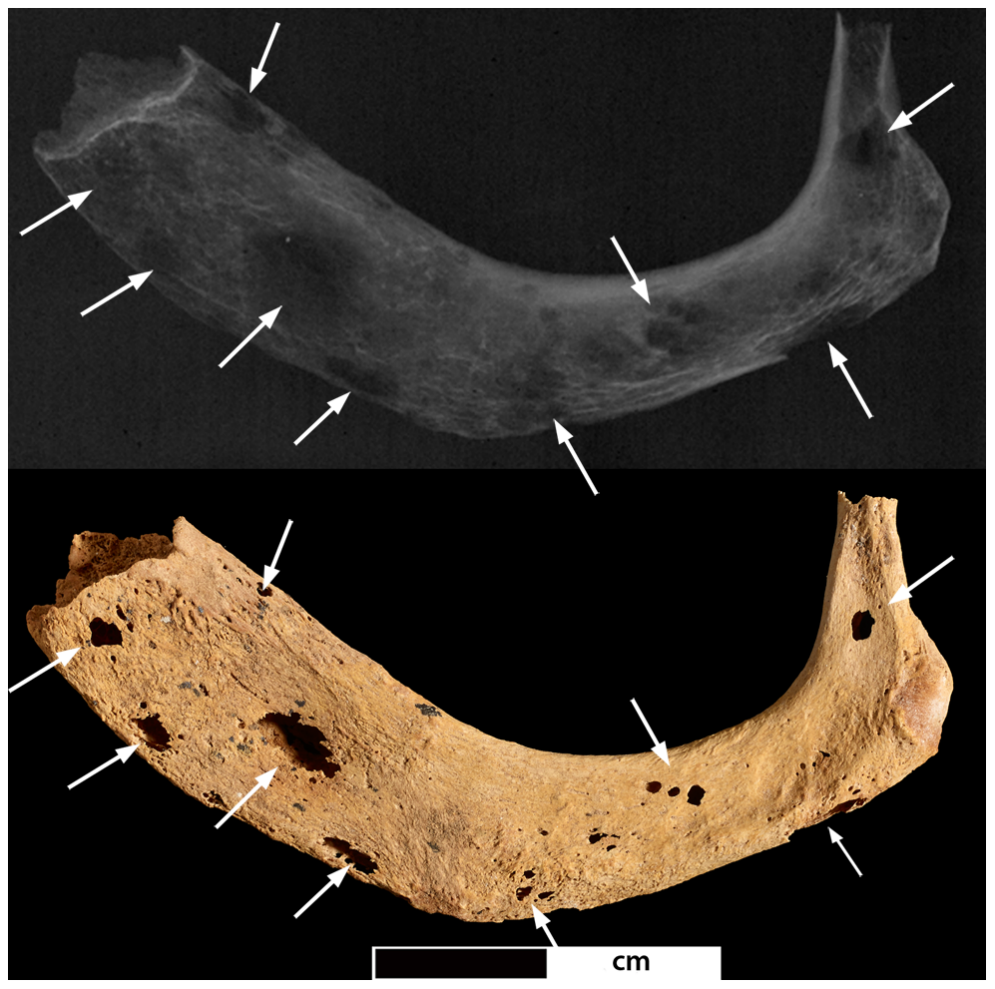
Lytic lesions in the first rib. Photo- and radiograph of the left first rib (superior surface. Arrows indicate location of the lesions.

All ribs on both sides display a large number of lesions, usually ranging between 2 and 4 mm in diameter. The vertebral ends and shafts are similarly affected. Due to the high degree of post-depositional fragmentation of the ribs, the exact amount of pathological changes cannot be established with precision. The most conspicuous lesions were observed in the shafts of the first ribs. An elliptical lesion with ragged edges (9×4 mm) is present on the superior side of the left first rib shaft. On the inferior side, the lesion had not fully penetrated the surface, and only minuscule perforations are present. A small focus of reactive periosteal new bone formation is visible overlying the opening, representing the only lesion associated with periosteal new bone formation (Figure 7). Upon radiographic examination, this lesion extends internally within the bone and is accompanied by another seven foci of 3–6 mm (Figure 8).

**Figure 9:**
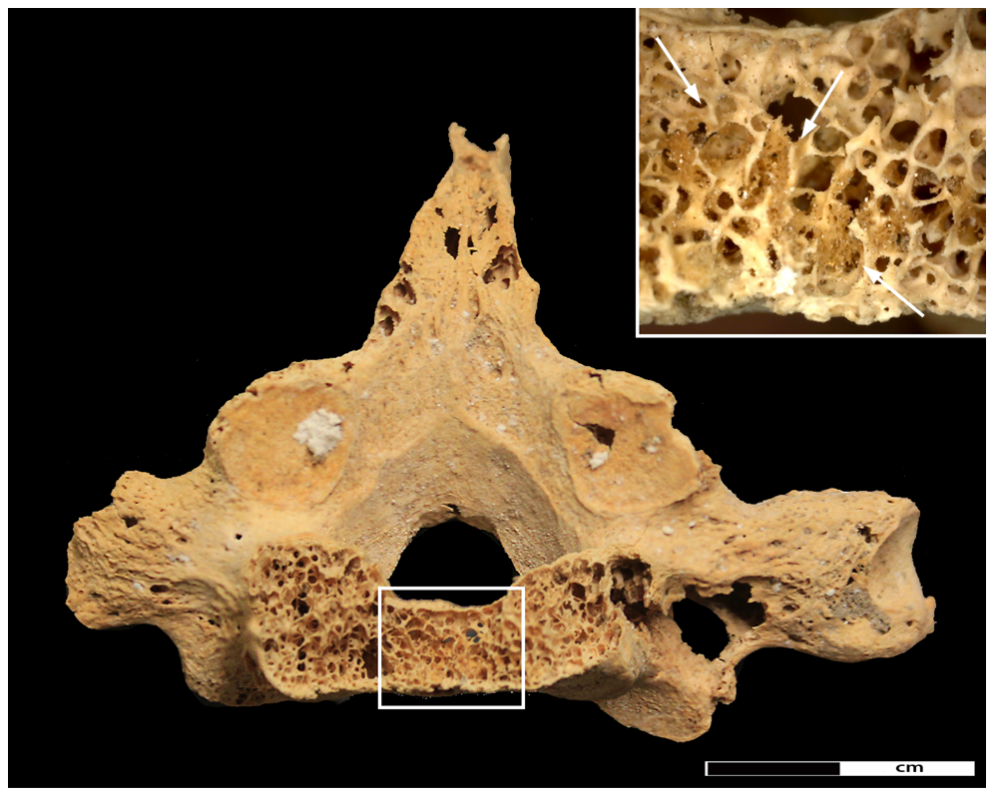
Destructive lesion in vertebral body of the 7^th^ thoracic vertebrae. Detail of the pathological changes in the 7^th^ thoracic vertebra. Rectangle indicates area of new bone infill of the spongiosa. Close-up of new bone formation indicated by arrows is shown in the insert; arrows indicate new bone formation.

**Figure 10:**
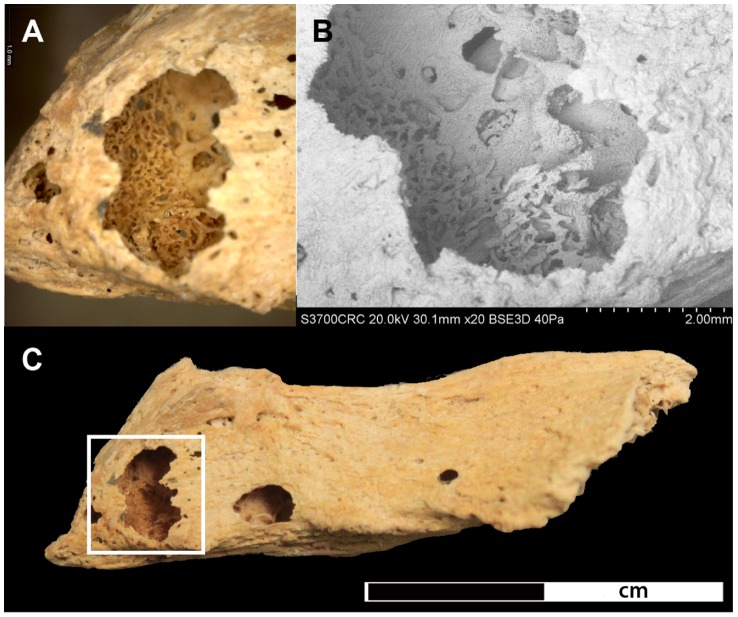
Lytic lesion in the spinous process of the 5^th^ thoracic vertebra. A) shows a close-up of bone formation at 35x magnification located within the lytic focus, B) SEM image of the lytic focus C) shows the complete spinous process with the location of the lytic focus highlighted in the rectangle.

Defining the size and shape of most lesions in the vertebrae was difficult due to extensive fragmentation caused by post-depositional damage in combination with the pathological changes. Only the cervical vertebrae were sufficiently preserved, displaying three to five sub-circular lesions in the vertebral bodies (5–8 mm) and neural arches (2–3 mm). The thoracic and lumbar sections of the spine were the most affected parts of the entire skeleton. Damage was particularly extensive in the vertebral bodies, with discernible lesions ranging in size from between 5 and 12 mm. The neural arches were similarly affected, displaying smaller sub-circular lesions (3–5 mm). A large lesion affecting almost the entire body of 7^th^ thoracic vertebra (Figure 9), as well as the spinous process of the 5^th^ thoracic vertebra feature new bone formation within the cancellous bone along the lesion margins (Figure 10).

**Figure 11:**
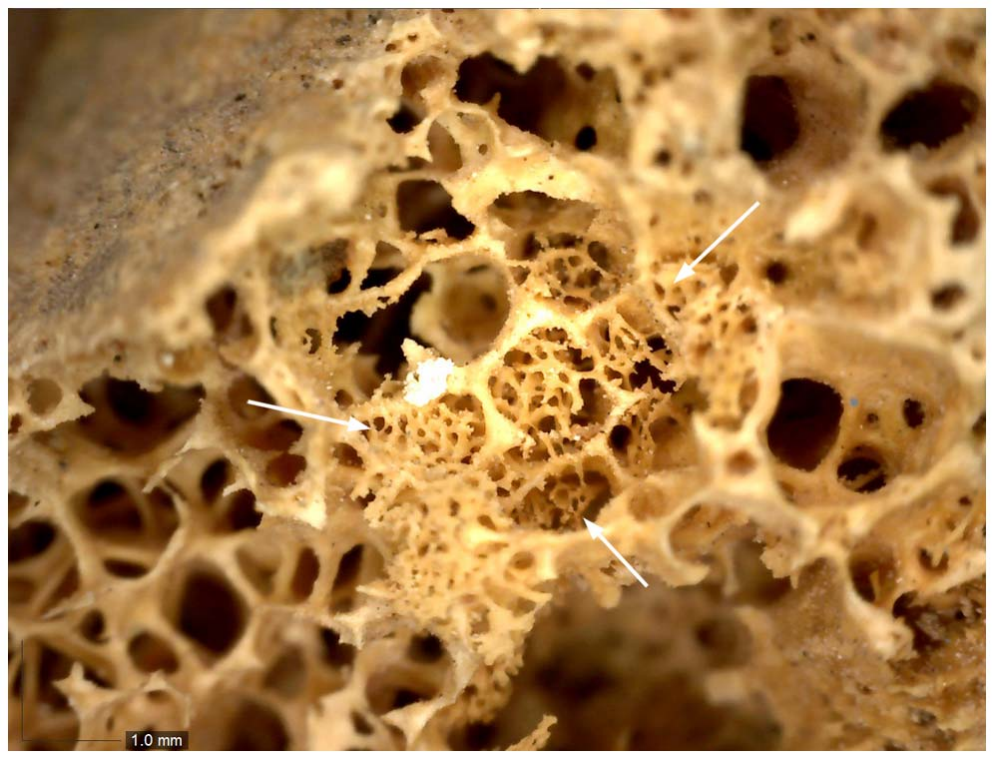
Detail of new bone formation in the iliac crest. The close-up shows a focus of new bone formation indicated by arrows in a lytic lesion in the iliac crest (40x magnification).

**Figure 12:**
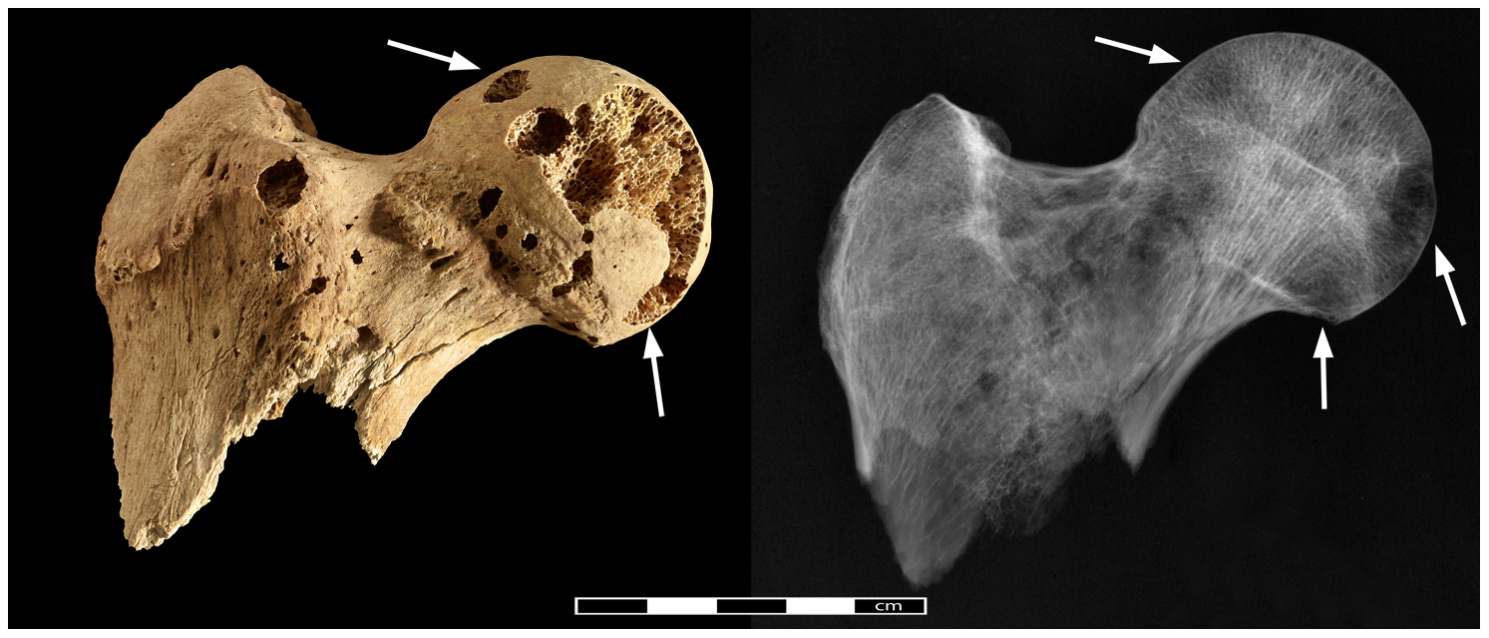
Pathological changes in the right femoral head. Photo- and radiograph of lytic lesions in the right femoral head (arrows indicate areas of pathological lesions).

Both innominate bones again suffered from heavy fragmentation. Within the fragments, distinctive circular cavitations within the cancellous bone were observed in the better preserved iliac crest and ischial tuberosity of both sides. Circular erosions were also present on the inner cortical side of the fragments of the iliac blades even though, due to heavy fragmentation, it is difficult to ascertain whether they are due to pathology or post-mortem damage. Formation of new bone within the trabecular structures was observed in one lesion in the right iliac crest (Ø 6.5 mm) (Figure 11) and one in the right ischium (Ø 7.5 mm). Despite post-mortem damage, several cavitations were observed on the femoral heads, with the right one displaying 7 cortical defects (5–8 mm) and a lesion on the anterior side of the greater trochanter (Figure 12).

## Discussion

Several differential diagnostic options can account for the observed changes in skeleton Sk244-8.

### Metastatic Carcinoma

Metastatic organ cancer is the most common source of metastasizing tumors affecting the skeleton [15]. Bone tissue is one of the preferential sites for metastatic expansion [63]. Tumor cells spread to the bones either through direct extension of a primary soft tissue tumor, through the lymphatic system, cerebro-spinal fluid or most importantly through haematogenous dissemination. Thus, predilected sites for metastasis formation are those bone structures rich in haematopoietic bone marrow and the vertebrae, pelvis, ribs, sternum, skull, clavicles, scapulae, humeral and femoral heads (in decreasing order of frequency) are the most commonly affected elements [63]. The elements distal to the elbow and knee are generally very rarely involved [17].

Skeletal response to metastatic tumours can either be osteolytic (75%), osteoblastic (15%) or a combination of both (10%) [17]. The type, distribution and density of metastatic lesions are dependent on the primary source of the tumour as well as on the duration of the disease. Carcinomas with purely osteolytic lesions are those of the thyroid, kidneys, adrenal glands, uterus and gastrointestinal tract. Exclusively osteosclerotic lesions are produced by carcinoma of the prostate gland. Mixed lesions can occur in carcinomas of the lung, breast, cervix, ovaries and testicles, though the ratio of formation to resorption is highly variable. However, none of these patterns is without exception [63]. In osteolytic lesions, bone resorption due to growth of tumour cells commences in cancellous bone characterised by internal scalloping. Only in advanced stages does destruction progress into the cortical bone [19]. The resulting lesions can vary considerably both in size and shape. Depending on the aggressiveness of the disease process, they can range between well circumscribed (geographic) to poorly defined (motheaten or permeative) bone destruction, the latter reflecting a more aggressive type [63], [64]. Due to the mechanism of metastasis formation, the number and size of lesions visible upon radiographic examination usually exceeds the number of lesions visible externally, making the radiographic appearance of lesions one of the key differential diagnostic features of metastatic carcinoma [64]. Single metastases are rare; in the majority of cases multiple lesions are present [15].

### Multiple Myeloma

Multiple myeloma is a neoplastic condition of the plasma cells of the bone marrow [15]. Originating in the haematopoietic marrow and cancellous structures of bone, multiple myeloma produces numerous destructive lesions that can be very similar to those caused by a carcinoma type cancer [64]. Differentiating between multiple myeloma and metastatic carcinoma in dry bone is considered a major challenge and may not always be possible [19], [28].The main differential diagnostic features are size and shape of the lesions. In contrast to metastatic carcinoma, the lesions are usually small, uniform in size, spherical with effaced edges, and are much denser and regular in distribution than carcinoma lesions. Suppressed osteoblast formation in tumorous foci is one of the hallmark pathophysiological features of multiple myeloma [65], [66]. Therefore, remodelling along the edges of lesions or new bone formation as is seen in the individual from Amara West is absent in multiple myeloma. [64].

### Mycosis

Several fungal infections can produce lytic lesions in the skeleton which may mimic the appearance of metastatic carcinoma [67] even though, with the exception of African histoplasmosis, skeletal involvement is generally rare [43: 217]. Bone infection occurs secondary to haematogenous dissemination. Skeletal lesions caused are almost exclusively lytic in nature with only little new bone formation [43: 213]. Key features in differentiating between metastatic carcinoma and fungal infections are the appearance of the lytic lesions and bone remodelling. In mycoses lytic lesions appear as fronts of resorption, in contrast to the space-occupying lesions produced by bone metastases. Newly formed bone is rare in fungal infections but if present it usually appears as characteristic blunt spiculae [67]. In contrast to metastatic carcinoma, skeletal lesions produced by mycotic infections occur throughout the skeleton and also affects the distal portions of the long bones and small hands of the hand and feet. The lesions observed in the individual from Amara West do not conform to the features associated with fungal infection, thus leaving it an unlikely differential diagnosis.

### Taphonomic damage

**Figure 13:**
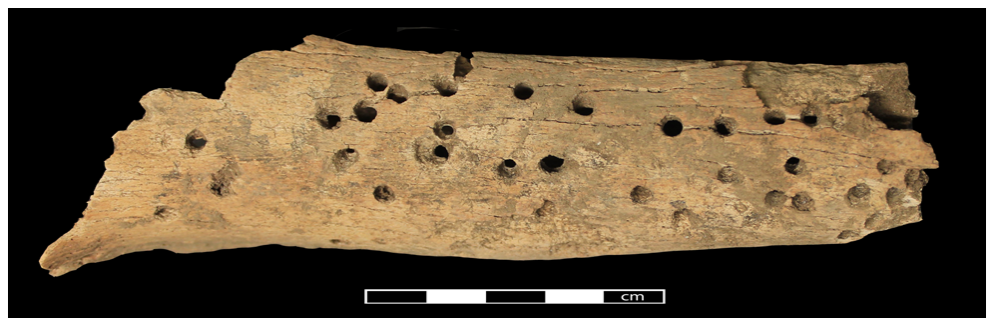
Post-mortem damage caused by insects. Tibia of a skeleton from Amara West showing extensive damage caused by dermestid beetles.

**Figure 14:**
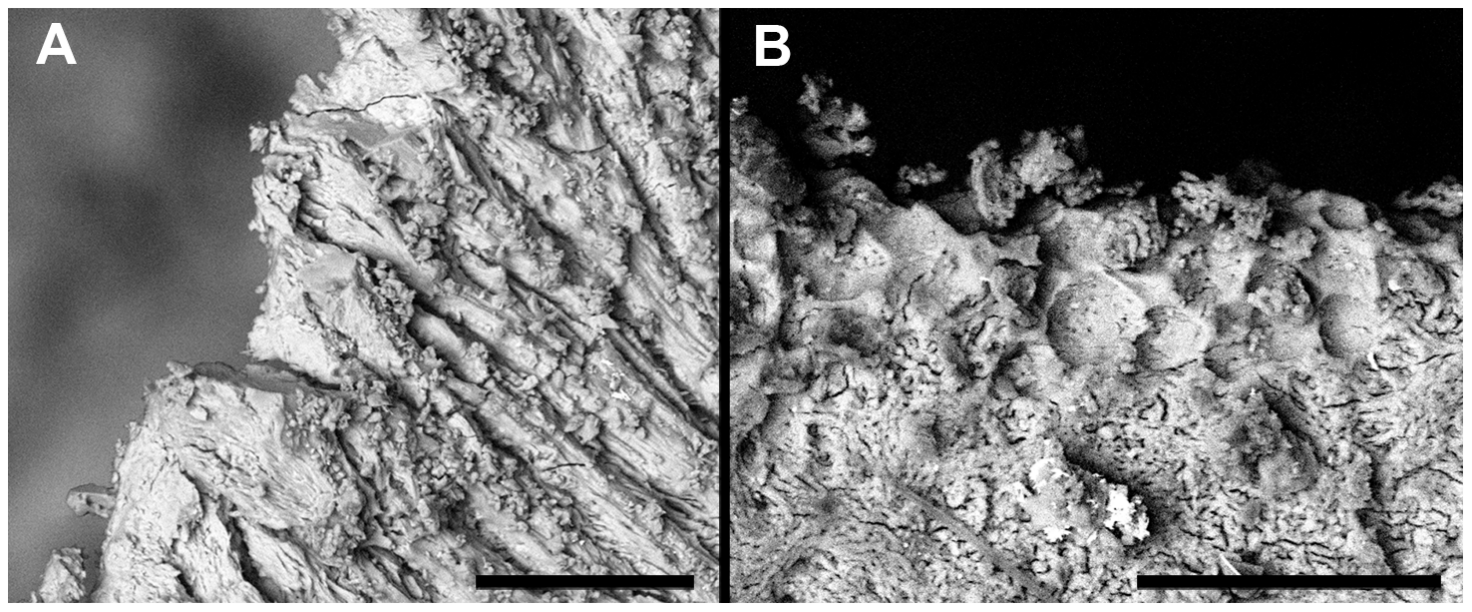
Comparison of SEM-images of taphonomic and pathological lesions. A) shows the margin of a defect caused by post-mortem damage. B) shows pathological changes on the margin of a lytic lesion in the cortical surface (bar on the bottom of each image indicates 100 µm).

Post-depositional processes could also produce damage to the bone mimicking the osteolytic lesions caused by a tumour [27]. Breakage and surface erosion are generally common problems in archaeological human remains. Small round holes similar to metastatic lesions can be caused by a variety of factors including roots, water, and termites [68] or dermatid beetles [69]. While root or water damage is uncommon at Amara West, destruction likely caused by osteophageous insects is frequently encountered (Figure 13). However, even though these holes may appear similar on the bone surface, they do not expand within the bone but rather appear to be regular punched out tunnels through the entire bone. In addition, insects do not show any preference for particular elements but, if present, tend to affect all parts of the skeleton. Further supporting evidence was obtained through targeted SEM analyses in order to better characterise the nature of the lesion margins. While in some examined lesions, post-mortem damage was established as the cause (Figure 14a), others show clear evidence of osteoclastic activity (Figure 14b). Therefore, even though some post-mortem damage is certainly present in the bones of Sk244-8, the radiographic appearance of the lesions, in combination with the observation of associated periosteal new bone formation, provides sufficient evidence to confirm that the majority of the observed lesions are indeed of pathological origin.

The size, shape and distribution of the irregularly shaped, often poorly defined to circular, lesions affecting the ribs, vertebrae, clavicle, scapulae, pelvis, sternum, humeral and femoral heads of skeleton 244-8 from Amara West are thus most probably the result of a metastatic carcinoma. The irregularly distributed lesions of varying shape and size as well as their subcortical appearance, strongly argue against ascribing them to multiple myeloma. The origin of the metastases, i.e. the primary tumour location, is not possible to ascertain. Based on the examination of dry bone alone, the origin of the metastases and the location of the primary tumour is difficult to ascertain, if not impossible [19]. Generally, organ cancers most commonly producing bone involvement are those of the breast and the prostate gland, followed by the lung, thyroid gland and kidneys [70]. While prostate cancer can be excluded based on the type of metastatic lesions, cancer of the lung and associated structures, or the thyroid gland, gastrointestinal tract or liver, are possible sites for the primary tumour. Breast cancer does occur in men [71], though rarely (<1% of cancers), thus remains a possible source of the lesions.

### Cancer in antiquity

This 25–35 year old man from Amara West, buried around 1200 BC, further evidence, provides another piece of evidence that cancer is in fact not a modern phenomenon [25], [29]. The apparent absence of cancer in archaeological remains may also partly be an illusion created by issues of bone preservation, and due to the fact that methods of analysis are inadequate to detect initial changes within bone. Due to financial, time and logistical reasons, human remains are usually not systematically radiographed, and bone metastases originating in cancellous tissue only penetrate the bone surface in their advanced stages. If the immune system was already compromised by other negative influences in a person’s life, people may not have survived long enough to develop full skeletal metastases. Thus, evidence for a large proportion of tumours could be missed when skeletal remains are analysed [72]. Another challenge in detecting cancer in ancient human remains is the poor preservation of bone which often prevents the clear identification of lytic lesions and precludes the diagnosis of incomplete remains [27]. With increasing numbers of skeletal collections and more detailed analysis, as well as more readily available standard radiographic equipment, the evidence for cancer in antiquity could increase significantly.

Recovered as part of an archaeological research project, this individual can be set within a cultural and historical framework, but also within the context of changing environmental conditions, and specific diet and subsistence strategies. The potential exists, therefore, to explore possible underlying causes of cancer in an ancient population, before the onset of modernity. As such it could provide important new insights into cancer aetiology and epidemiology in the past. Even though today’s leading causes of cancer are all products of modern living conditions and industrialisation, a large number of environmental carcinogens also occur naturally (e.g. asbestos), and would have affected our ancestors in the same way [73]. The carcinogenic effects of smoke from wood fires, particularly when indoors are well known [1], [74]. The houses at Amara West typically feature hearths, but also bread ovens [50], often within roofed spaces without windows, where smoke would have dissipated slowly. In modern Sudan, the common usage of fires in poorly ventilated rooms of small mudbrick huts is still considered one of the major factors leading to lung cancer [75]; similar conditions are common across early societies, including those where other evidence of cancer has been identified [25]. Bitumen, known also to cause cancer in individuals occupationally exposed to bitumen fumes [76] was already used by ancient Egyptians for waterproofing or embalming [77].

Infectious diseases can also lead to cancer. Schistosomiasis has plagued inhabitants of Egypt and Nubia since at least 1500BC [78], [79], [80] and is now recognized as a common cause of bladder cancer [81]. In addition, it has been associated with an increased risk of male breast cancer as a consequence of the hormonal disturbances resulting from liver cirrhosis secondary to schistosomiasis infection [82]. This may also account for the fact that the male to female breast cancer ratio in Egypt today is far greater than anywhere else in the world [83]. Detecting evidence for schistosomiasis infection in ancient human remains can be achieved through immunobiological testing of mummified soft tissue [84] or secondary through recovery of parasite eggs from soil samples from graves or latrines as well as from coprolites [78]. Despite attempts to find parasite evidence in a selected number of soil samples from other graves, no such evidence has yet been identified at Amara West. Even though the disease is absent from the Amara West area today, palaeoenvironmental data suggests that the habitat would likely have been favourable for sustaining populations of gastropods transmitting schistosomiasis during the time of New Kingdom occupation. Though unproven, an underlying schistosomiasis infection leading to breast cancer in the man from Amara West as well as other Nubian and Egyptian individuals [46] represents a plausible cause. The link between gastrointestinal cancer and infection by *helicobacter pylori*, which is recognized to have affected human populations since prehistory [85] is also well established [86]. The individual from Amara West, only 25–35 years old at death, further underlines that cancer was restricted to neither old age nor elite social status. From a modern clinical point of view, the young age-at-death of the man from Amara West may seem unusual for the onset of skeletal metastases, but it remains unknown whether the underlying causes of cancer affected people in the same way and at the same speed as they do today.

The lack of evidence for cancer in antiquity may to a large extent, be the result of reduced life expectancy, and thus less time to develop skeletal lesions if the immune system is already compromised by an inadequate supply of nutrients and diseases. This represents one of the major problems in inferring the absence or presence of disease in the past in general [87]. The archaeological and historical record certainly provides plenty of evidence for possible causes of developing cancer. Despite recent advances, the genetic background for cancer predisposition is still far from being understood today [88], [89]. Even though it may perhaps remain unknown, there is no reason to assume that predisposing genetic factors were not present in the past. The man from Amara West does indicate that it was indeed possible to develop skeletal lesions of cancer, provides a glimpse into one individual’s life experience, and cautions against claims for the absence, or presence, of any disease based on skeletal evidence alone.

## Conclusions

Dating to *c*. 1200 BC, the individual from Amara West in Sudan represents one of the earliest people identified in the bioarchaeological record anywhere in the world, who suffered secondary malignant neoplasm. This provides further support for the claim that cancer is a disease of considerable antiquity. It may not have been as prevalent as today, and there is little doubt that the main factor accounting for the increased prevalence of cancer is undoubtedly modern living. Environmental carcinogens deriving from wood smoke (amongst others) have been present in the human living environment for a long time, yet cancer is still very rare in the palaeopathological record. However, with increasing numbers of human remains available for study, and technical advances and better availability of analytical equipment, in combination with increased attention to detail by those conducting palaeopathological research the number of ancient individuals with cancer may increase significantly. Increasing scientific research on mummified remains facilitated through improved and more readily available imaging techniques such as computed tomography-scanning, will provide an additional dataset, allowing for detection of cancer in the soft tissues of preserved bodies. Gaining a better understanding of the disease’s history and epidemiology in the past may significantly contribute to further investigate and understand the underlying mechanisms leading to cancer today.

Increasingly, scholars working in palaeopathology are thinking more about the value of using their data to contribute to knowledge of disease today [90]. It is well known that people with cancer today are developing resistance to the chemotherapy used, similar to antibiotic resistance to the treatment of infectious disease [10], and there have also been recommendations for future research into the evolution of cancer to improve the management of this common disease. For example, by using biomolecular approaches to individual skeletons and mummies with evidence of cancer (ancient DNA analysis), it might be possible to show changes in their genome which can be mapped against the very evolution of the human population, and detect mutations in specific genes that are known to be associated with particular types of cancer. In turn, and by linking these data to contextual considerations i.e. the living environment that the person experienced, it may be possible to understand better why and in what ways cancer therapies need to be developed. Furthermore, exploring the reasons for a population’s susceptibility to developing cancer may be aided by examining the nature of the immune system of peoples affected by cancer in the past, via biomolecular analysis, to see how cancer may have changed throughout the long history of evolution. Research in these areas [91], [92] has indicated the potential for this approach. Consequently, by taking an evolutionary approach to cancer, this knowledge may prove a crucial element in finding ways to address one of the world’s major health problems of the 21^st^ century.

The presence of the disease in pre-modern populations further raises questions about the theoretical concept of epidemiological transitions undergone by human populations [46]. Even though cancer represents one of the key features of the 2^nd^ epidemiological transition, its presence in a pre-2^nd^ transitional population highlights the fact that cancer as such is not only linked to longevity but also to infectious diseases. The boundaries between the 1^st^ and 2^nd^ transition may not be as clear-cut as previously thought [46].

## References

[1] Boyle P, Levin P, editors (2008) World Cancer Report 2008. Lyon: International Agency for Research on Cancer.

[2] WHO (2013) Fact sheet N°297: Cancer.

[3] David AR, Zimmerman MR (2010) Cancer: an old disease, a new disease or something in between? Nature Reviews: Cancer 10: 728–733. 20814420 10.1038/nrc2914

[4] McKeown RE (2009) The Epidemiologic Transition: Changing Patterns of Mortality and Population Dynamics. American Journal of Lifestyle Medicine 3: 19S–26S. 20161566 10.1177/1559827609335350 PMC2805833

[5] Karpozilos A, Pavlidis N (2004) The treatment of cancer in Greek antiquity. European Journal of Cancer 40: 2033–2040. 15341975 10.1016/j.ejca.2004.04.036

[6] Nunn JF (2002) Ancient Egyptian Medicine. Norman: University of Oklahoma Press.

[7] Breasted JH (1930) The Edwin Smith Surgical Papyrus: Hieroglyphic transliterations, translations and commentary. Chicago: University of Chicago Press.

[8] Sanchez GM, Meltzer ES (2012) The Edwin Smith Papyrus: Updated Translation of the Trauma Treatise and Modern Medical Commentaries: Updated Translation of the Trauma Treatise and Modern Medical Commentaries. Atlanta: Lockwood Press.

[9] Mukherjee S (2010) The Emperor of All Maladies: A Biography of Cancer: Scribner.

[10] Stearns SC (2012) Evolutionary medicine: its scope, interest and potential. Proceedings of the Royal Society of Biological Science 279: 4305–4321. 10.1098/rspb.2012.1326 PMC3479795 22933370

[11] Nesse RM, Williams GC (1994) Why we get sick. The new science of Darwinian medicine. New York: Vintage Books.

[12] Manderson L (2011) Anthropologies of cancer and risk uncertainty and disruption. In: Singer M, Erickson PI, editors. A companion to medical anthropology 1st Edition. Chichester, West Sussex: Blackwell Publishing Limited. pp. 323–338.

[13] Brown PJ, Armelagos GJ, Maes KC (2011) Humans in a world of microbes: the anthropology of infectious diseases. In: Singer M, Erickson PI, editors. A companion to medical anthropology 1st Edition. Chichester, West Sussex: Blackwell Publishing Limited. pp. 253–270.

[14] Buikstra J, Roberts C, editors (2012) The Global History of Paleopathology: Oxford University Press.

[15] Dorfman HD, Czerniak B (1998) Bone Tumors. St. Louis: Mosby, Inc.

[16] Bertram JS (2000) The molecular biology of cancer. Molecular Aspects of Medicine 21: 167–223. 11173079 10.1016/s0098-2997(00)00007-8

[17] Greenspan A, Remagen W (1998) Differential Diagnosis of Tumors and Tumor-like Lesions of Bones and Joints. Philadelphia, New York: Lippincott-Raven.

[18] Barrett R, Kuzawa CW, McDade T, Armelagos GJ (1998) Emerging and Re-emerging Infectious Diseases: The Third Epidemiologic Transition. Annual Reviews in Anthropology 27: 247–271.

[19] Ortner DJ (2003) Identification of Pathological Conditions in Human Skeletal Remains. London: Academic Press. 645 p.

[20] Franceschi S, Wild CP (2013) Meeting the global demands of epidemiologic transition – The indispensable role of cancer prevention. Molecular Oncology 7: 1–13. 23218182 10.1016/j.molonc.2012.10.010 PMC5528406

[21] Cox M (2000) Ageing Adults from the Skeleton. In: Cox M, Mays S, editors. Human Osteology: In Archaeology and Forensic Science. London: Greenwich Medical Media. pp. 61–81.

[22] Chamberlain A (2006) Demography in Archaeology. Cambridge: Cambridge University Press. 235 p.

[23] Gabler K (2009) Die Medja - dein Lieferant und Helfer. Untersuchungen zu medja von Deir el-Medine anhand von Ostraka und Papyri [in German, unpublished master thesis]. München: Ludwig-Maximilians-Universität

[24] Parkin TG (2003) Old Age in the Roman World: A Cultural and Social History. Baltimore: John Hopkins University Press.

[25] Capasso LL (2005) Antiquity of Cancer. International Journal of Cancer 113: 2–13. 15389511 10.1002/ijc.20610

[26] Doll R, Peto R (1981) The Causes of Cancer: Quantitative Estimates of Avoidable Risks of Cancer in the United States Today. Journal of the National Cancer Institute 66: 1192–1308. 7017215

[27] Brothwell DR (2012) Tumors: Problems of Differential Diagnosis in Paleopathology. In: Grauer AL, editor. A Companion to Paleopathology. Oxford: Wiley-Blackwell. pp. 420–433.

[28] Marks MK, Hamilton MD (2007) Metastatic Carcinoma: Palaeopathology and Differential Diagnosis. International Journal of Osteoarchaeology 17: 217–234.

[29] Nerlich AG, Rohrbach H, Bachmeier B, Zink A (2006) Malignant tumors in two ancient populations: An approach to historical tumor epidemiology. Oncology Reports 16: 197–202. 16786146

[30] Strouhal E, Kritscher H (1990) Neolithic case of a multiple myeloma from Mauer (Vienna, Austria). Anthropologie 28: 78–97.

[31] Gladykowska-Rzeczycka J (1991) Tumors in antiquity in East and Middle Europe. In: Ortner DJ, Aufderheide AC, editors. Human Paleopathology - Current Syntheses and Future Options. Washington, London: Smithsonian Institution Press. pp. 251–256.

[32] Rokhlin D (1966) Disease in ancient man. Moskov: Nauka Ed.

[33] Strouhal E (2001) Malignant tumours in past populations in Middle Europe In: La Verghetta M, Capasso L, editors. Proceesings of the XIIIth European Meeting of the Paleopathology Association. Teramo: Edigrafical. pp. 265–272.

[34] Strouhal E (1976) Tumors in the remains of Ancient Egyptians. American Journal of Physical Anthropology 45: 613–620. 793419 10.1002/ajpa.1330450328

[35] Baker BJ, Judd M (2012) Development of Paleopathology in the Nile Valley. In: Buikstra J, Roberts C, editors. The Global History of Paleopathology: Oxford University Press. pp. 209–234.

[36] Pahl WM (1986) Tumors of bone and soft tissue in ancient Egypt and Nubia: a synopsis of the detected cases. International Journal of Anthropology 1: 267–275.

[37] Strouhal E, Vyhanek L (1981) New cases of malign tumours from Late Period cemeteries at Abusir and Saqqara (Egypt). Ossa 8: 165–189.

[38] Strouhal E (1993) A case of metastatic carcinoma from Christian Sayala (Egyptian Nubia). Anthropologischer Anzeiger 51: 97–115. 8333739

[39] Strouhal E, Vyhnanek L (1982) New cases of malignant tumors from late period cemeteries at Abusir and Saqqara (Egypt). Ossa 8: 165–189.

[40] Strouhal E (1991) A case of primary carcinoma from Christian Sayala (Egyptian Nubia). Journal of Paleopathology 3: 51–65. 8333739

[41] Wells C (1963) Ancient Egyptian Pathology. Journal of Laryngology and Otology 77: 261–265.

[42] Prates C, Sousa S, Oliveira C, Ikram S (2011) Prostate metastatic bone cancer in an Egyptian Ptolemaic mummy, a proposed radiological diagnosis. International Journal of Paleopathology 1: 98–103. 29539324 10.1016/j.ijpp.2011.09.002

[43] Aufderheide AC, Rodríguez-Martín C (1998) The Cambridge Encyclopaedia of Human Paleopathology. Cambrigde, New York: Cambridge University Press. 578 p.

[44] Ho JHC (1972) Nasopharyngeal Carcinoma. In: Klein G, Weinhouse S, editors. Advances in Cancer Research. London: Academic Press, Inc. pp. 57–92.

[45] Aufderheide AC (2003) The Scientific Study of Mummies. Cambridge: Cambridge University Press.

[46] Esche E, Mummert A, Robinson J, Armelagos GJ (2010) Cancer in Egypt and Nubia. Anthropologie 48: 33–39.

[47] Dupras T, de Voogt A, Francigny V, Williams L, Lacey J (2014) Advanced Metastatic Carcinoma in the Paleopathological Record: A Case Study from the Sudan. 41th Annual Meeting of the Paleopathology Association. Calgary, Alberta, Canada.

[48] Spencer P (1997) Amara West I. The architectural report. London: The Egypt Exploration Society.

[49] Spencer N (2012) Insights into Life in occupied Kush during the New Kingdom: New Research at Amara West. Der Antike Sudan 23: 21–28.

[50] Spencer N (forthcoming) Amara West: considerations on urban life in occupied Kush. In: Welsby D, Anderson JR, editors. Proceedings of the 12th International Conference for Nubian Studies. Leuven: OLA.

[51] Binder M (2011) The 10th-9th century BC - New Evidence from Cemetery C of Amara West. Sudan & Nubia 15: 39–53.

[52] Binder M, Spencer N, Millet M (2011) Cemetery D at Amara West: the Ramesside Period and its aftermath. British Museum Studies in Ancient Egypt and Sudan.

[53] Spencer N, Macklin MG, Woodward JC (2012) Reassessing the abandonment of Amara West: the impact of a changing Nile? Sudan & Nubia 16: 37–43.

[54] Ryan P, Cartwright C, Spencer N (2012) Archaeobotanical research in a pharaonic town in ancient Nubia. The British Museum Technical Research Bulletin 6: 97–107.

[55] Binder M, Spencer N (In Press) The bioarchaeology of Amara West in Nubia: Investigating the impacts of political, cultural and environmental change on health and diet. In: Fletcher A, Antoine D, Hill JD, editors. Regarding the Dead. London: British Museum Press.

[56] Van Pelt WP (forthcoming) Revising Egypto-Nubian Relationss in New Kingdom Lower Nubia: From Egyptianization to Cultural Entanglement. Cambridge Archaeological Journal 23.

[57] Smith ST (2003) Wretched Kush. London, New York: Routledge.

[58] Buikstra JE, Ubelaker DH (1994) Standards for Data Collection from Human Remains. Lafayetteville, Arkansas: Arkansas Archaeological Survey. 206 p.

[59] Brickley M, McKinley JI, editors (2004) Guidelines to the Standards for Recording Human Remains. Reading: Institute of Field Archaeologists Paper Number 7.

[60] Bruzek J (2002) A method for visual determination of sex, using the human hip bone. American Journal of Physical Anthropology 117: 157–168. 11815949 10.1002/ajpa.10012

[61] Brooks S, Suchey JM (1990) Skeletal age determination based on the os pubis: a comparison of the Acsádi-Nemeskéri and Suchey-Brooks methods. Human Evolution 5: 227–238.

[62] Scheuer L, Black S (2000) Developmental juvenile osteology. San Diego: Academic Press.

[63] Resnick D (1995) Diagnosis of Bone and Joint Disorders. St. Louis, MO: W. B. Saunders.

[64] Rothschild BM, Hershkovitz I, Dutour O (1998) Clues Potentially Distinguishing Lytic Lesions of Multiple Myeloma From Those of Metastatic Carcinoma. American Journal of Physical Anthropology 105: 241–250. 9511917 10.1002/(SICI)1096-8644(199802)105:2<241::AID-AJPA10>3.0.CO;2-0

[65] Yaccoby S (2010) Advances in the understanding of myeloma bone disease and tumour growth. British Journal of Haematology 149: 311–321. 20230410 10.1111/j.1365-2141.2010.08141.x PMC2864366

[66] Sezer O (2009) Myeloma Bone Disease: Recent Advances in Biology, Diagnosis, and Treatment. The Oncologist 14: 276–283. 19286761 10.1634/theoncologist.2009-0003

[67] Hershkovitz I, Rothschild BM, Dutour O, Greenwald C (1998) Clues to recognition of fungal origin of lytic skeletal lesions. American Journal of Physical Anthropology 106: 47–60. 9590524 10.1002/(SICI)1096-8644(199805)106:1<47::AID-AJPA4>3.0.CO;2-A

[68] Huchet JB, Deverly D, Gutierrez B, Chauchat C (2011) Taphonomic Evidence of a Human Skeleton Gnawed by Termites in a Moche-Civilisation Grave at Huaca de la Luna, Peru. International Journal of Osteoarchaeology 21: 92–102.

[69] Huchet JB, Le Mort F, Rabinovich R, Blau S, Coqueugniot H, et al (2013) Identification of dermestid pupal chambers on Southern Levant human bones: inference for reconstruction of Middle Bronze Age mortuary practices. Journal of Archaeological Science 40: 3793–3803.

[70] Layer D (2005) Skelettmetastasen. In: Freyschmidt J, Stäbler A, editors. Handbuch diagnostische Radiologie - Muskoloskelettales System 2: Springer. pp. 327–338.

[71] Anderson WF, Jatoi I, Tse J, Rosenberg PS (2010) Male Breast Cancer: A Population-Based Comparison With Female Breast Cancer. Journal of Clinical Oncology 28: 232–239. 19996029 10.1200/JCO.2009.23.8162 PMC2815713

[72] Rothschild BM, Rothschild C (1995) Comparison of Radiologic and Gross Examination for Detection of Cancer in Defleshed Skeletons. American Journal of Physical Anthropology 97: 357–363. 7604891 10.1002/ajpa.1330960404

[73] Hueper WC (1963) Environmental Carcinogenesis in Man and Animals. Annals of the New York Academy of Sciences 108: 963–1038. 14081526 10.1111/j.1749-6632.1963.tb13433.x

[74] Delgado J, Martinez LM, Sanchez TT, Ramirez A, Iturria C, et al (2005) Lung cancer pathogenesis associated with wood smoke exposure. Chest 128: 124–131. 16002925 10.1378/chest.128.1.124

[75] Awadelkarim KD, Mariani-Costantini R, Elwali NE (2012) Cancer in the Sudan: An overview of the current status of knowledge on tumor patterns and risk factors. Science of the Total Environment 423: 214–228. 21071068 10.1016/j.scitotenv.2010.09.010

[76] Binet S, Pfohl-Leszkowicz A, Brandt H, Lafontaine M, Castegnaro M (2002) Bitumen fumes: review of work on the potential risk to workers and the present knowledge on its origin. Science of the Total Environment 300: 37–49. 12685469 10.1016/s0048-9697(02)00279-6

[77] Serpico M, White R (2000) Resins, amber and bitumen. In: Nicholson PT, Shaw I, editors. Ancient Egyptian Materials and Technology. Cambridge: Cambridge University Press.

[78] Bouchet F, Harter S, Le Bailly M (2003) The State of the Art of Paleoparasitological Research in the Old World. Memórias do Instituto Oswaldo Cruz 98: 95–101. 12687768 10.1590/s0074-02762003000900015

[79] Miller RL, Armelagos GJ, Ikram S, De Jonge N, Krijger FW, et al (1992) Palaeoepidemiology of schistosoma infection in mummies. British Medical Journal 304: 355–356. 10.1136/bmj.304.6826.555 PMC1881415 1559065

[80] Reyman TA, Zimmerman MR, Lewin PK (1977) Autopsy of an Egyptian mummy. 5. Histopathologic investigation. Canadian Medical Association Journal 117: 470–472. 332304 PMC1879978

[81] Sitas F, Parkin DM, Chirenje M, Stein L, Abratt R, et al (2008) Part II: Cancer in Indigenous Africans - causes and control. Lancet Oncology 9: 786–795. 18672214 10.1016/S1470-2045(08)70198-0

[82] Buzdar AU (2003) Breast cancer in men. Oncology (Williston Park) 17: 1361–1364. 14606362

[83] Mustacchi P (2003) Schistosomiasis. In: Kufe DW, Pollock RE, Wechselbaum RR, editors. Holland-Frei, Cancer Medicine 6th edition. Hamilton (ON): BC Decker.

[84] Hibbs CA, Secor WV, Van Gerven D, Armelagos GJ (2011) Irrigation and infection: The immunoepidemiology of schistosomiasis in ancient Nubia. American Journal of Physical Anthropology 145: 290–298. 21469072 10.1002/ajpa.21493

[85] Linz B, Balloux F, Moodley Y, Manica A, Liu H, et al (2007) An African origin for the intimate association between humans and Helicobacter pylori. Nature 445: 915–918. 17287725 10.1038/nature05562 PMC1847463

[86] Polk DB, Peek RM (2010) Helicobacter pylori: gastric cancer and beyond. Nat Rev Cancer 10: 403–414. 20495574 10.1038/nrc2857 PMC2957472

[87] Wood JW, Milner GR, Harpending HC, Weiss KM (1992) The Osteological Paradox - Problems of Inferring Prehistoric Health from Skeletal Samples. Current Anthropology 33: 343–370.

[88] Bartsch H, Dally H, Popanda O, Risch A, Schmezer P (2007) Genetic risk profiles for cancer susceptibility and therapy response. Recent Results in Cancer Research 174: 19–36. 17302182 10.1007/978-3-540-37696-5_2

[89] Frank SA (2004) Genetic predisposition to cancer - insights from population genetics. Nature Reviews: Genetics 5: 764–772. 10.1038/nrg1450 15510167

[90] Zuckerman MK, Turner BL, Armelagos GJ (2012) Evolutionary thought in paleopathology and the rise of the biocultural approach. In: Grauer A, editor. A companion to paleopathology. Oxford: Wiley Blackwell.

[91] Fornaciari G, Marchetti A, Pellegrini S, Ciranni R (1999) K-ras mutation in the tumour of King Ferrante I of Aragon (1431–1494) and environmental mutagens at the Aragonese court of Naples. International Journal of Osteoarchaeology 9: 302–306.

[92] Schlott T, Eiffert H, Schmidt-Schultz T, Gebhardt M, Parzinger H, et al (2007) Detection and analysis of cancer genes amplified from bone material of a Scythian royal burial in Arzhan near Tuva, Siberia. Anticancer Research 27: 4117–4119. 18225581

[93] Strouhal E (1994) Malignant Tumors in the Old World. Paleopathology Newsletter 85 (supplement): 1–6.

[94] Strouhal E (1991) Myeloma Multiplex versus Osteolytic Metastatic Carcinoma: Differential Diagnosis in Dry Bones. International Journal of Osteoarchaeology 1: 219–224.

[95] Strouhal E (1978) Ancient Egyptian case of carcinoma. Bulletin of the New York Academy of Medicine 54: 290–302. 343851 PMC1807435

[96] Schultz M, Parzinger H, Posdnjakov DV, Chikisheva TA, Schmidt-Schultz TH (2007) Oldest known case of metastasizing prostate carcinoma diagnosed in the skeleton of a 2,700-year-old Scythian King from Arzhan (Siberia, Russia). International Journal of Cancer 121: 2591–2595. 17918181 10.1002/ijc.23073

